# Anticancer Activity of Ag(I) N-Heterocyclic Carbene Complexes Derived from 4,5-Dichloro-1H-Imidazole

**DOI:** 10.1155/2008/384010

**Published:** 2008-07-03

**Authors:** Doug A. Medvetz, Khadijah M. Hindi, Matthew J. Panzner, Andrew J. Ditto, Yang H. Yun, Wiley J. Youngs

**Affiliations:** ^1^Knight Chemical Laboratory 105, Department of Chemistry, The University of Akron, Akron, OH 44325-3601, USA; ^2^Olsen Research Center 301D, Department of Biomedical Engineering, The University of Akron, Akron, OH 44325-0302, USA

## Abstract

A class of Ag(I) N-heterocyclic carbene silver complexes, **1**–**3**, derived from 4,5-dichloro-1H-imidazole has been evaluated for their anticancer activity against the human cancer cell lines OVCAR-3 (ovarian), MB157 (breast), and Hela (cervical). Silver complexes **1**–**3** are active against the ovarian and breast cancer cell lines. A preliminary in vivo study shows **1** to be active against ovarian cancer in mice. The results obtained in these studies warrant further investigation of these compounds in vivo.

## 1. INTRODUCTION

Research efforts into the development of new chemotherapeutic anticancer agents play an important role in the future treatment of cancer. The divergence from platinum-based agents is becoming a point of emphasis with the goal being to find drugs that are as effective as platinum without the severity of side effects. Metallocene dichlorides and dirhodium carboxylates have shown anticancer activity, however neither class of compounds has demonstrated sufficient effectiveness to pursue past phase II clinical trials [1, 2].

Recently, silver complexes have been reported to have anticancer activity in vitro. Egan has reported that silver complexes of coumarin derivatives possess anticancer activity against certain types of cancer [3]. Zhu has reported that silver carboxylate dimers possess anticancer activity against human carcinoma cells [4]. McKeage has shown phosphine complexes of silver to be active anticancer agents, even against cisplatin resistant cell lines [5].

Research into using silver is essential as toxicity is believed to be quite low. In fact, silver has been detected in 29 human tissues in trace amounts; however, there is no known physiological function for silver. In vitro studies have shown that silver salts do have an effect on dermal fibroblasts; however, the effect usually does not lead to cell death [6]. Also, silver is being used in vivo to coat foreign materials. Artificial heart valves, along with cardiac and urinary catheters, are being coated with silver in medical applications to reduce or prevent the infection rate of various microbes [7–10]. The problem with ingestion of silver is a permanent discoloration of the skin known as argyria. However, argyria is not believed to be harmful to the body physically and it takes an excessive consumption of silver to develop this condition [11].

Based on the previously discussed reports of the antitumor activity of silver and our expertise in silver N-Heterocyclic carbene complexes [12–15], along with other reported N-Heterocyclic carbene complexes possessing anticancer activity [16–18], we have begun to examine the anticancer activity of Ag(I) complexes of N-Heterocyclic carbenes. Herein, we report the anticancer activity of three Ag(I)-N-Heterocyclic carbene complexes derived from 4,5-dichloro-1H-imidazole. These silver complexes appear to be stable to light and water making them viable candidates for use as chemotherapeutic agents.

## 2. RESULTS AND DISCUSSION

The imidazolium salts and their silver acetate complexes **1**, **2**, and **3** were synthesized similarly to procedures in a previously published procedure from our group [19]. These silver complexes have been shown to be very stable and can be synthesized efficiently [19].

Silver complexes of type **1**, **2**, and **3** have been chosen over the use of silver salts because of their stability. The use of silver salts in vivo is not practical because the free silver ions will form complexes with salts in the bloodstream.

The MTT assay was run against the cancer cell lines OVCAR-3 (ovarian), MB157 (breast), and Hela (cervical) to determine the in vitro anticancer efficacy of **1–3**, the imidazolium salt precursors, silver nitrate, and silver acetate relative to that of Cisplatin. Cancer cells were plated at cell densities of 5000 cells per well in 96-well plates and allowed to incubate overnight. The following day, the test compounds were dissolved in DMSO [20] and diluted into the respective cell culture media to the desired micromolar concentrations. The media in the wells was replaced with fresh media containing test compound or DMSO for control, and the cells were incubated for 72 hours. Following this test, period MTT protocol was followed by adding 10 *μ*L of MTT in PBS to each well, and the plates were incubated for four hours. In viable, metabolically active cells, MTT is reduced in the mitochondria by the enzyme succinate dehydrogenase forming insoluble bluish purple formazan crystals. These formazan crystals are then resolublized by addition of 100 *μ*L of SDS in dilute HCl. The optical density of each well is then read at 570 nm. The imidazolium salts showed no activity against the cancer cell lines, while the silver complexes **1–3** were very efficacious against the ovarian and breast cancer cell lines, while showing very minimal effect on the cervical cancer cell line. The IC_50_ concentrations for active compounds, where IC_50_ stands for the concentration that causes a 50% reduction in cell viability, are reported in Table 1.

**Figure 2:**
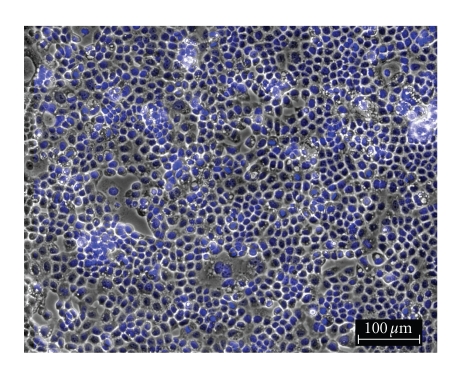
OVCAR-3 control.

**Figure 3:**
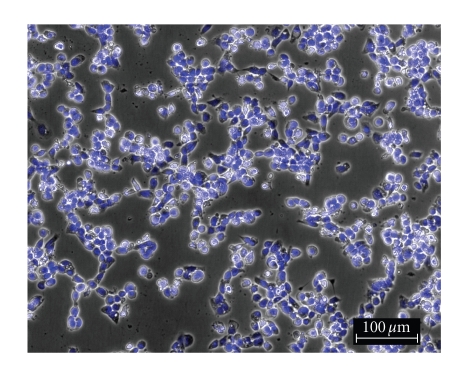
OVCAR-3 incubated with Cisplatin.

**Figure 4:**
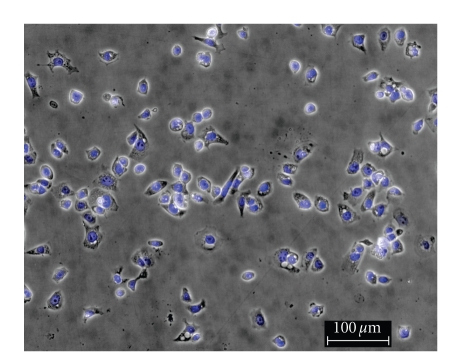
OVCAR-3 incubated with **1**.

**Figure 5:**
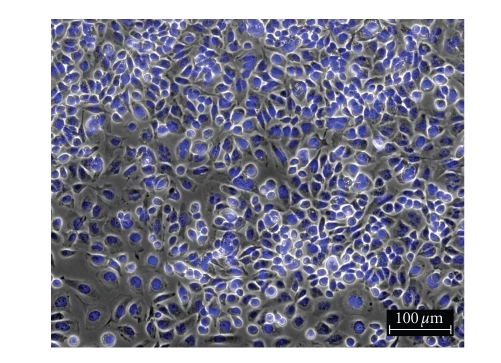
MB157 control.

**Figure 6:**
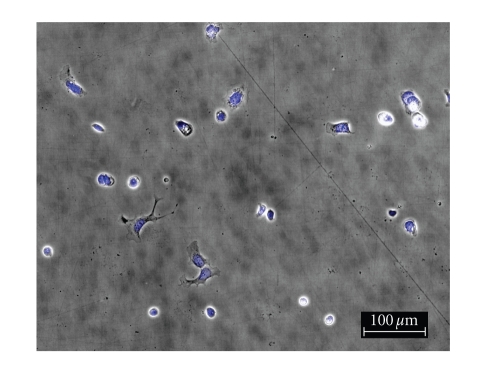
MB157 incubated with Cisplatin.

**Figure 7:**
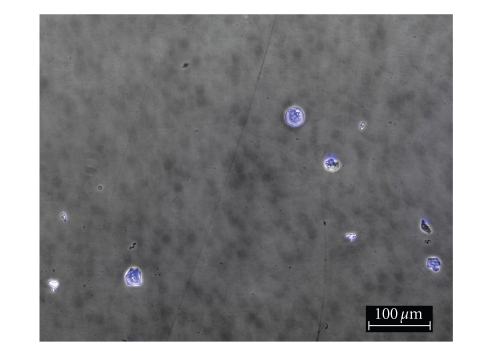
MB157 incubated with **1**.

To determine the effect of silver complexes **1–3** and Cisplatin on the morphology of the OVCAR-3 and MB157 cells in culture, phase contrast pictures were taken of tested cell culture. The cells were dyed with Hoesch for clarity purposes only. Cells were plated in 24-well plates and allowed to grow to confluency. Test compounds were dissolved in DMSO and diluted into the respective cell culture media to the desired micromolar concentrations. The media in the wells was replaced with fresh media containing test compound or DMSO for control. Cells were incubated with test compounds at 50 *μ*M for 36 hours. Selected results are shown as pictures in Figures 2–7.

The above described pictures show that these silver compounds have significant effect on cell viability. These results qualitatively show that both the OVCAR-3 and MB157 cell lines did not survive the exposure to **1–3** at 50 *μ*M. All of the silver complexes demonstrated superior effectiveness as compared to Cisplatin at the same concentration.

**Figure 8:**
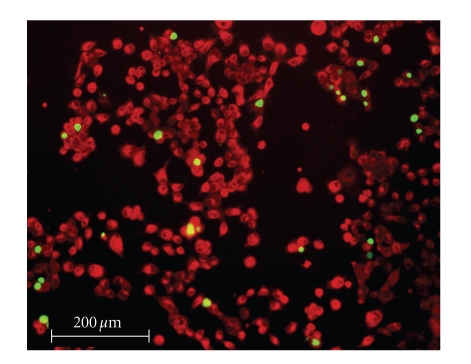
OVCAR-3 control.

**Figure 9:**
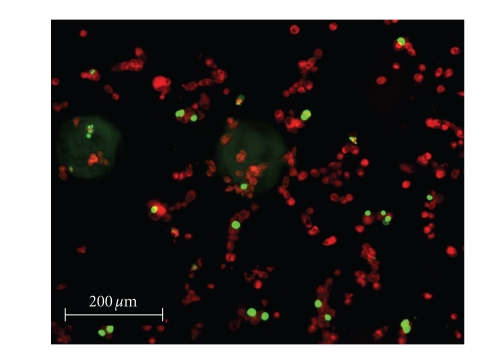
OVCAR-3 incubated with Cisplatin.

**Figure 10:**
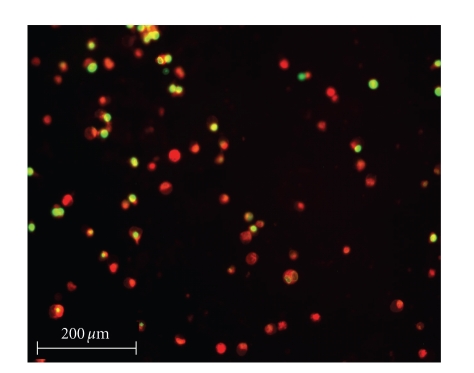
OVCAR-3 incubated with **1**.

**Figure 11:**
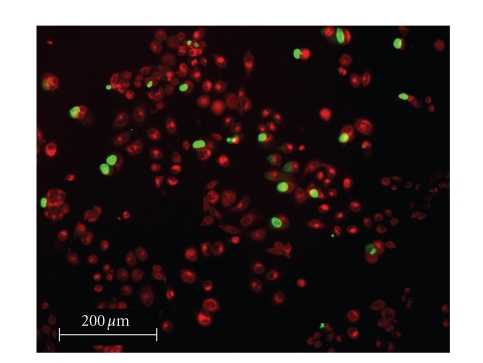
MB157 control.

**Figure 12:**
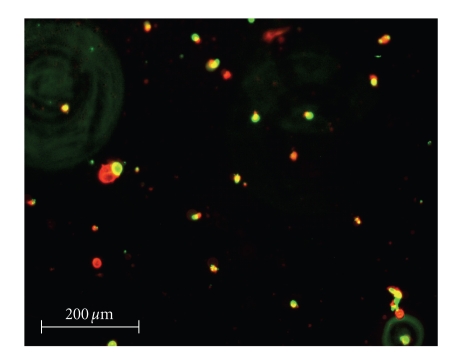
MB157 incubated with Cisplatin.

**Figure 13:**
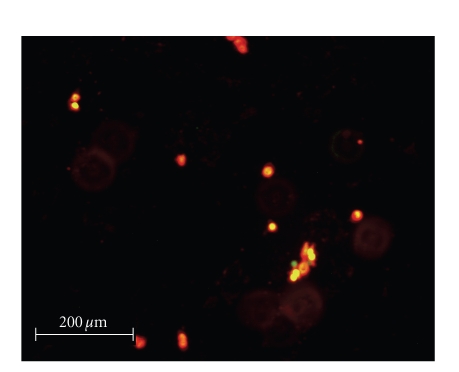
MB157 incubated with **1**.

To quantify the efficacy of the silver complexes, a live/dead cell assay was performed. Selected fluorescence images are shown in Figures 8–13. Cells were plated in 24-well plates and allowed to grow to confluency. Test compounds were dissolved in DMSO and diluted into the respective cell culture media to the desired micromolar concentrations. The media in the wells was replaced with fresh media containing test compound or DMSO for control. Cells were incubated with test compounds at 50 *μ*M for 36 hours. Live cells fluoresced red due to their metabolic activity that converted the stain C_12_-resazurin into fluorescent C_12_-resorufin. Dead cells accumulated the Sytox green stain since their cell membranes had been compromised, resulting in green fluorescence. The silver complexes **1–3** produced significantly higher death among the OVCAR-3 cell compared to Cisplatin and control OVCAR-3 cells (*P* < .0001). The viabilities of OVCAR-3 cells exposed to **1–3** were 11%, 0%, and 0%, respectively. Meanwhile, OVCAR-3 cells exposed to Cisplatin resulted in 78% viability, which was not significantly different than the control cells 93% viability (*P* = .5579). All silver complexes and Cisplatin produced significant death among MB157 breast cancer cells. The live/dead assay revealed 10% cell viability for MB157 cells exposed to Cisplatin, which was significantly different than the 92% viability for the MB157 control cells (*P* < .0001). Based on the live/dead assay, **1–3**, showed significantly superior cell lysing capabilities with ovarian cancer compared to Cisplatin. These silver complexes also were shown to completely kill the breast cancer cell line MB157. Our findings suggest silver complexes of this type may be excellent candidates for therapeutic drugs for certain types of cancer.

**Figure 14:**
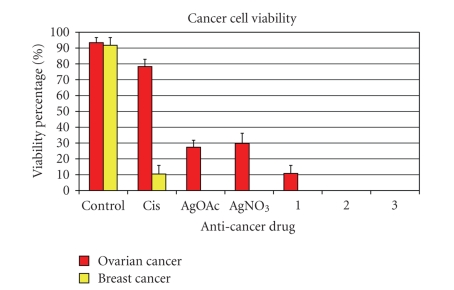
Percent viability of OVCAR-3 and MB157 after testing with cisplatin (Cis), silver acetate, (AgOAc), and silver nitrate (AgNO3), 1, 2, and 3. The percentages are based on cell counts from the live/dead assay data after cells were tested for 36 hours. Cells were incubated with test compounds at 50 *μ*M.

The cell viability percentages were tallied from the live/dead assay data and shown in Figure 14. The data from microscopy and live/dead assay coincides with the MTT assay except for the Cisplatin data. This discrepancy can be attributed to the fact that Cisplatin takes longer incubation periods to be fully effective against the cells lines. In the MTT, assay cells had to be incubated for 72 hours to see maximum efficacy, here the incubation time was only 36 hours.

**Figure 15:**
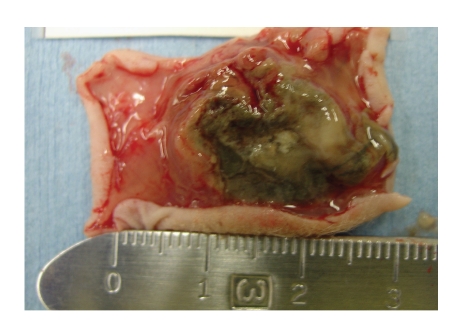
Necrotic tumor mass.

**Figure 16:**
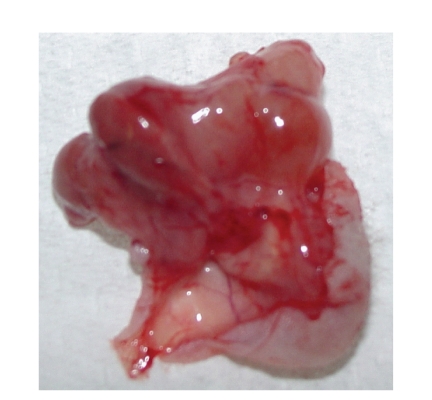
Normal tumor mass.

**Figure 17:**
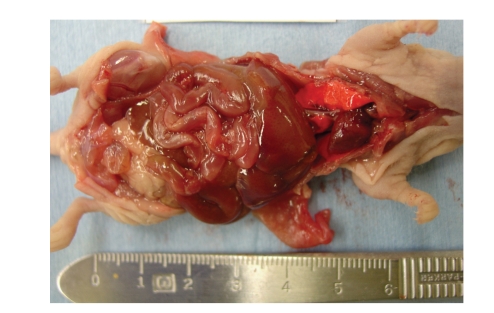
View of the internal organs.

Due to the activity of these silver complexes, in vitro, a preliminary in vivo study was run to determine if **1** was active against an ovarian cancer xenograft model. Ten million OVCAR-3 cells were injected subcutaneously into the back of female athymic nude mice. Upon visible tumor growth, approximately six weeks, silver complex **1** was injected subcutaneously at the tumor site every third day for ten days. Each dose consisted of 333 mg/kg of **1** for a total of 1000 mg/kg over the ten day period. The high dose was chosen because a parallel study was being run, where IP injections were given at a dose of 100 mg/kg per day and it seemed relevant to give the same total dose over this ten-day period. Injections were only given every third day because the compound absorbed more slowly in the subcutaneous study. The mice were then necropsied to determine what effect **1** had on the tumors, as well as, on the internal organs. Selected pictures of the tumors, along with the internal organs, are shown in Figures 15–17. According to pathological results, **1** caused major cell death of the tumors, however, showed no ill-effects to the major organs of the mice.

## 3. CONCLUSION

In conclusion, we have shown a class of Ag(I) N-Heterocyclic carbenes to possess anticancer activity, in vitro, against ovarian (OVCAR-3) and breast (MB157) cancer; however, these silver complexes have little effect on cervical (Hela) cancer. Each compound, **1–3**, seems to be more active against the breast cancer cell line MB157 with IC_50_'s of around 10 *μ*M.

Preliminary in vivo studies show that **1** is active against an ovarian cancer xenograft model by subcutaneous injection. Further in vivo studies are underway to determine the effect of **1** on the ovarian cancer xenograft model, where the mode of delivery is through intraperitoneal injection. These findings indicate that N-Heterocyclic carbene silver complexes may be useful in cancer chemotherapy depending on the type of cancer.

## 4. EXPERIMENTAL

### 4.1. General

4,5-dichloro-1H-imidazole was purchased from TCI (Wellesly Hills, Ma, USA). 1-bromohexane was purchased from Aldrich (Louis Street, Mo ,USA). 2-bromomethylnaphthalene was purchased from Alfa (Ward Hill, Ma, USA). All chemicals were used without further purification. All mass spectrometry was performed at CCIC at The Ohio State University. ^1^H and ^13^C NMR spectra were obtained on a Varian 300 MHz or Varian 500 MHz instrument. All elemental analyses were performed at the University of Illinois at Urbana-Champaign. Pathology reports from the mouse study were performed at BioReliance.

### 4.2. Cell lines

The human cancer cell lines OVCAR-3 and MB157 were purchased from ATCC (Manassas, Va, USA). The OVCAR-3 cell line was grown in RPMI 1640 media with 2 mM L-glutamine and modified to contain 10 mM HEPES, 1 mM sodium pyruvate, 4.5 g/L glucose, and 1.5 g/L sodium bicarbonate and was supplemented with .01 mg/mL bovine insulin and 20% fetal bovine serum. The cells were grown at 37°C with 5% CO_2_ and passed every 2-3 days. The MB157 cell line was grown in DMEM with 4 mM L-glutamine modified to contain 4.5 g/L glucose and 1.5 g/L sodium bicarbonate and supplemented with 10% fetal bovine serum. The cells were grown at 37°C with 5% CO_2_ and passed every 2-3 days. The human cancer cell line Hela S_3_ was donated by Dr. Yang Yun of the Biomedical Engineering department at the University of Akron. The Hela cell line was grown in DMEM/F-12K media supplemented with 1% antibiotic/antimicotic and 10% fetal bovine serum. The cells were grown at 37°C with 5% CO_2_ and passed once a week.

### 4.3. MTT assay

The MTT assay was purchased from Molecular Probes (Part of Invitrogen). The respective cell lines were grown to confluency and plated in 96-well plates at 5000 cells per well in triplicate and allowed to incubate overnight. Compounds **1**, **2**, **3**, the imidazolium salts precursors, Cisplatin, silver acetate, and silver nitrate were dissolved in DMSO to a concentration of 0.1 M and diluted into cell culture media to the desired testing concentrations. Media in each well was removed and replaced with the fresh media containing test compounds. The test compounds were allowed to incubate for 24, 48, or 72 hours after which the MTT protocol was followed. A stock solution of MTT was prepared by adding 1 mL of PBS to the preweighed vial containing MTT. 10 *μ*L of this stock solution were added to each well and allowed to incubate for 4 hours. A stock solution of sodium dodecyl sulfate was prepared by adding 10 mL of 0.01 M HCl to the preweighed SDS vial. Following the 4 hour incubation period, 100 *μ*L of the SDS : HCl solution were added and incubated overnight. The optical density was read at 570 nm on Molecular Devices Spectramax M2 plate reader.

### 4.4. Microscopy

Cells were plated at 50 000 cells per well in 24-well plates and the culture was allowed to grow to confluency. Compounds **1**, **2**, **3**, and Cisplatin were dissolved in DMSO to a concentration of 0.1 M and diluted into cell culture media to the desired testing concentrations. Media in each well was removed and replaced with the fresh media containing test compounds. The test compounds were incubated for 36 hours. Afterwards, cells were rinsed with PBS and fixed for 10 minutes with freshly prepared 0.5% formaldehyde solution. After fixation, cells were rinsed and permeabilized with 0.2% Triton X solution for 10 minutes. Hoechst nuclear dye was prepared according to the manufacturer's recommendations and carefully applied to the cells. After 30 minutes of incubation, cells were rinsed three times with PBS and visualized with fluorescence microscopy. All images were captured using AxioVision 200 by Zeiss with a 10x Plan Neofluor Zeiss objective and a high-resolution CCD HRm camera. The flourescent and phase contrast images were colocalized using the AxioVision software version 4.6.

### 4.5. Live/dead assay

The live/dead assay was purchased from Invitrogen (Carlsbad, Ca, USA). Cells were plated in 24-well plates. Compounds **1**, **2**, **3**, and Cisplatin were dissolved in DMSO to a concentration of 0.1 M and diluted into cell culture media to the desired testing concentrations. Media in each well was removed and replaced with the fresh media containing test compounds. The test compounds were allowed to incubate for 36 hours. The live/dead stains were then prepared by dissolving 40 *μ*g of C_12_-Resazurin in 100 *μ*L of DMSO as a 1 mM stock solution. A 50 *μ*M C_12_-Resazurin solution was then made up by diluting 2.5 *μ*L of the stock solution into 47.5 *μ*L of DMSO. The Sytox green solution was prepared by diluting 5 *μ*L of a 10 *μ*M stock solution in 45 *μ*L of DMSO. The staining solution was prepared by diluting 48 *μ*L of 50 *μ*M C_12_-Resazurin and 48 *μ*L of Sytox green stain into 4.704 mL of 1X PBS for final concentrations of 500 nM. The cell media was then removed from culture, and the cells were washed with 1X PBS. Cells were then covered with 200 *μ*L of live/dead staining solution and incubated for 15 minutes at room temperature protected from light. Lastly, the cells were visualized using a fluorescent microscope. Three random images were taken per well, and the channels were combined, and the images were processed using Axiovision software as previously described. Cell viabilities were found to be significant using Tukey's multiple comparison among means (*α* = 0.05).

### 4.6. Ovarian cancer xenograft model

Three-week-old female athymic nude mice were purchased from Harlan (Indianapolis, In, USA) and housed for one week prior to the experiment. Ten million cells were injected subcutaneously into the back of three animals, right below the ear. The animals were monitored and the weights were recorded 2-3 times per week until the tumors grew to be visible (macroscopic tumors grew in about six weeks). The next day, and every third day following for ten days, the animals were injected with a dose of 333 mg/kg of compound **1** subcutaneously at the tumor site for a total of 1000 mg/kg. After ten days, the animals were sacrificed, and a full necropsy was performed. The brain, liver, lungs, heart, kidneys, spleen, and tumors were sent for pathology report. Synthetic, NMR, and crystallographic data for the imidazolium salts and their silver acetate structures are available as Supplementary Material at doi:10.1155/2008/384010.

## Supplementary Material

The synthesis and characterization of silver complexes **1–3**, along with their respective imidazolium salts, are available as supplementary information. This includes detailed synthesis and values for NMR, mass spec, elemental analysis, and some X-ray crystallography.

## Figures and Tables

**Scheme 1:**
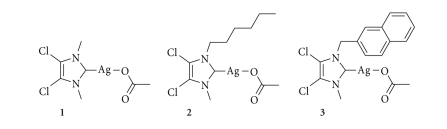


**Figure 1:**
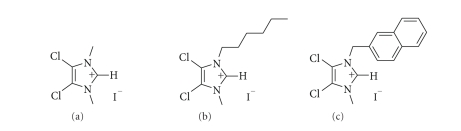
Imidazolium salts precursors of (a) **1**, (b) **2**, (c) **3**.

**Table 1:** IC_50_ concentrations of silver drugs.

	OVCAR-3^(a)^	MB157^(a)^	Hela^(a)^
Cisplatin	12	25	25
AgNO_3_	35	5	50
AgOAc	20	12	NA
**1**	35	8	>200
**2**	30	20	>200
**3**	20	10	>200

^(a)^IC_50_ concentrations are reported in micromolar. NA = not achievable due to solubility of AgOAc.
