# Supplementary material for: Anticancer Activity of Ag(I) N-Heterocyclic Carbene Complexes Derived from 4,5-Dichloro-1H-Imidazole
Source: Met Based Drugs. 2008 Jul 3;2008:384010. doi: 10.1155/2008/384010 (PMC2443426; doi:10.1155/2008/384010)
Supplement: Supplementary file 1 — The synthesis and characterization of silver complexes 1–3, along with their respective imidazolium salts, are available as supplementary information. This includes detailed synthesis and values for NMR, mass spec, elemental analysis, and some X-ray crystallography. [file 384010.f1.pdf]

Synthesis of 1-methyl-4,5-dichloroimidazole (a). 4,5-Dichloroimidazole (1.23 g, 9.00 mmol) and potassium hydroxide (2.24 g, 40.0 mmol) were stirred in acetonitrile (50 mL) for 2 h at room temperature. The excess KOH was filtered from the solution and iodomethane (0.562 mL, 9.00 mmol) was added. The reaction mixture was stirred at room temperature for 24 h. The volatile components were removed and the crude product was redissolved in dichloromethane. The solid, presumable KI, was filtered and discarded and the volatile components were removed in vacuo to yield a yellow crystalline solid. Yield: (1.32 g, 8.72 mmol, 97%); Mp: 56-58 ˚C. Anal. Calc. for $C_4H_4Cl_2N_2$: C, 31.82; H, 2.67; N, 18.55. Found: C, 32.31; H, 2.74; N, 17.51. ESI-MS (m/z): calc., 157.0, $[M + Li]^+$; found, 156.9. ^{1}H NMR (300 MHz, DMSO-d_6): δ 3.60 (s, 3H, CH_3), 7.76 (s, 1H, NCHN). ^{13}C {^{1}H} NMR (75 MHz, DMSO-d_6): δ 32.3 (NCH_3), 136.2 (NCHN), 124.0 (C-Cl), 112.6 (C-Cl).

Synthesis of 1,3-dimethyl-4,5-dichloroimidazolium iodide (A). Compound **a** (1.32 g, 8.72 mmol) was dissolved in acetonitrile (30 mL) and excess iodomethane (2 mL, 32.1 mmol) was added. The mixture was refluxed at 85 ˚C for 2 d. The volatile components were removed in vacuo to yield a green crystalline solid. Yield: (2.45 g, 8.37 mmol, 96%); Mp: 179-182 ˚C. Anal. Calc. for $C_5H_7Cl_2N_2I$: C, 20.50; H, 2.41; N, 9.56. Found: C, 20.31; H, 2.27; N, 9.22. ESI-MS (m/z): calc., 165.0, $[C_5H_7Cl_2N_2^+]$; found, 164.9. ^{1}H NMR (300 MHz, DMSO-d_6): δ 3.83 (s, 6H, CH_3), 9.42 (s, 1H, NCHN). ^{13}C {^{1}H} NMR (75 MHz, DMSO-d_6): δ 35.0 (NCH_3), 136.6 (NCHN), 118.9 (C-Cl).

1-hexyl-3-methyl-4,5-dichloroimidazolium iodide (B). KOH (2.24 g, 0.040 mol) and 4,5-dichloro-1H-imidazole (1.23 g, 0.009 mol) were added to 50 mL of CH_3CN and stirred for 2h. The excess KOH was filtered off and 1-bromohexane (1.26 mL, 0.009 mol) was added and the solution was refluxed for 24h. The solution was allowed to cool to RT and the KBr precipitate

was filtered off. Methyl iodide (2.5 mL, 0.036 mol) was added via syringe and the solution was refluxed overnight. The CH_3CN was removed in vacuo yielding an oily product. The oil was stirred in Et_2O resulting in a yellow powdery solid (2.20 g, 67%). The anion of **B** was exchanged from iodide to tetraphenyl borate and then x-ray quality crystals were grown from a concentrated acetonitrile solution. ^1H NMR (300MHz d_6-DMSO) δ 0.87 (t), 1.29 (s), 1.76 (m), 2.07 (s),

3.82 (s), 4.2 (t), 9.46 (s). ^{13}C NMR $\{^1H\}$ (75MHz, d_6-DMSO) δ 13.81, 21.80, 24.93, 28.19, 30.49, 35.04, 38.67, 48.25, 117.99, 119.29, 136.31. ESI-MS m/z 235. Anal. Calcd. for $C_{10}H_{17}N_2Cl_2I$ Theoretical: C, 33.08; H, 4.72; N, 7.72. Found: C, 32.10; H, 4.66, N, 7.41.

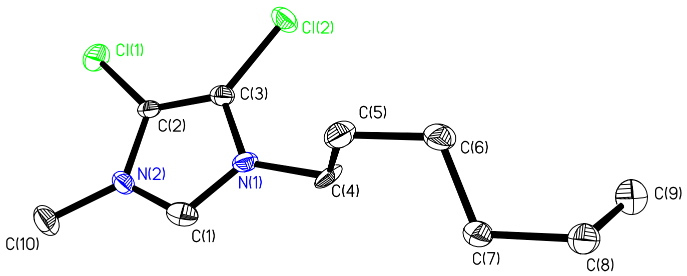

Crystal Structure of B. Thermal ellipsoids are at 50% probability. Hydrogen atoms and anion removed for clarity.

Table 1. Crystal data and structure refinement for **B**.

Identification code	dm	
Empirical formula	C68 H64 B2 Cl4 N4	
Formula weight	1100.71	
Temperature	100(2) K	
Wavelength	0.71073 Å	
Crystal system	Triclinic	
Space group	P1	
Unit cell dimensions	a = 9.851(4) Å	α= 82.567(6)°.
	b = 11.653(4) Å	β= 79.668(6)°.
	c = 13.614(5) Å	γ = 78.645(6)°.
Volume	1500.1(10) Å^3	

Z	2
Density (calculated)	1.218 mg/m^3
Absorption coefficient	0.242 mm^{-1}
F(000)	578
Crystal size	0.19 x 0.12 x 0.06 mm^3
Theta range for data collection	1.53 to 28.33°.
Index ranges	-12<=h<=12, -15<=k<=14, -17<=l<=17
Reflections collected	12915
Independent reflections	11704 [R(int) = 0.0224]
Completeness to theta = 28.33°	90.5 %
Absorption correction	Semi-empirical from equivalents
Max. and min. transmission	0.986 and 0.5785
Refinement method	Full-matrix least-squares on F^2
Data / restraints / parameters	11704 / 3 / 734
Goodness-of-fit on F^2	1.175
Final R indices [I>2sigma(I)]	R1 = 0.0656, wR2 = 0.1481
R indices (all data)	R1 = 0.0690, wR2 = 0.1502
Absolute structure parameter	0.11(5)
Largest diff. peak and hole	0.544 and -0.435 e.Å^{-3}

Table 2. Atomic coordinates (x 10^4) and equivalent isotropic displacement parameters ($Å^2$x 10^3) for dm. U(eq) is defined as one third of the trace of the orthogonalized U^{ij} tensor.

	x	y	z	U(eq)
Cl(1)	4055(1)	7482(1)	3463(1)	27(1)
Cl(2)	5833(1)	9710(1)	3853(1)	24(1)
Cl(3)	4258(1)	7835(1)	10491(1)	29(1)
Cl(4)	4446(1)	10314(1)	11664(1)	33(1)
N(1)	7641(3)	7688(3)	4129(2)	16(1)
N(2)	6545(3)	6316(3)	3923(2)	17(1)
N(3)	3435(3)	9685(3)	9198(2)	23(1)
N(4)	3557(4)	11210(3)	9916(2)	24(1)
C(1)	7721(4)	6538(3)	4121(3)	22(1)
C(2)	5655(4)	7384(3)	3794(3)	16(1)
C(3)	6326(4)	8228(3)	3929(3)	16(1)
C(4)	8765(4)	8284(4)	4286(3)	19(1)
C(5)	8593(4)	8572(4)	5368(3)	23(1)
C(6)	9616(4)	9362(4)	5483(3)	23(1)
C(7)	11157(4)	8803(4)	5183(3)	24(1)
C(8)	12190(4)	9615(4)	5234(3)	27(1)
C(9)	12073(5)	10692(4)	4463(4)	33(1)
C(10)	6230(5)	5153(4)	3845(3)	27(1)
C(11)	3323(4)	2877(3)	6968(3)	17(1)
C(12)	2656(4)	2040(4)	6683(3)	21(1)
C(13)	1280(4)	1911(4)	7082(3)	27(1)
C(14)	518(5)	2631(4)	7811(3)	34(1)
C(15)	1138(5)	3460(4)	8124(3)	29(1)
C(16)	2514(4)	3572(4)	7712(3)	22(1)
C(17)	5287(4)	4296(3)	6552(3)	18(1)
C(18)	4278(4)	5291(4)	6363(3)	24(1)
C(19)	4543(5)	6428(4)	6293(3)	30(1)
C(20)	5815(5)	6640(4)	6418(3)	32(1)
C(21)	6874(5)	5665(4)	6572(3)	28(1)
C(22)	6610(4)	4535(4)	6636(3)	22(1)
C(23)	5830(4)	1994(3)	7350(3)	18(1)

C(24)	6075(4)	790(4)	7234(3)	26(1)
C(25)	6655(5)	-61(4)	7913(4)	33(1)
C(26)	7042(5)	261(5)	8760(4)	37(1)
C(27)	6838(5)	1439(5)	8901(4)	36(1)
C(28)	6237(4)	2278(4)	8219(3)	25(1)
C(29)	5537(4)	2605(3)	5408(3)	16(1)
C(30)	4678(4)	2782(3)	4669(3)	20(1)
C(31)	5203(5)	2599(3)	3670(3)	25(1)
C(32)	6630(5)	2254(4)	3360(3)	30(1)
C(33)	7505(5)	2058(4)	4073(3)	29(1)
C(34)	6977(4)	2217(3)	5075(3)	20(1)
C(35)	3240(5)	10848(4)	9105(3)	26(1)
C(36)	3864(4)	9288(4)	10106(3)	22(1)
C(37)	3928(4)	10240(4)	10553(3)	23(1)
C(38)	3150(4)	8965(4)	8474(3)	24(1)
C(39)	1702(5)	8634(4)	8760(4)	34(1)
C(40)	1458(6)	7803(5)	8069(5)	48(1)
C(44)	3463(5)	12447(4)	10079(3)	33(1)
C(45)	-77(4)	6279(3)	1743(3)	16(1)
C(46)	-1532(4)	6480(3)	1733(3)	20(1)
C(47)	-2309(4)	7603(4)	1592(3)	25(1)
C(48)	-1680(4)	8583(4)	1470(3)	26(1)
C(49)	-256(5)	8427(4)	1485(3)	26(1)
C(50)	522(4)	7302(3)	1599(3)	19(1)
C(51)	2480(4)	4987(3)	1333(3)	19(1)
C(52)	2660(4)	5265(3)	297(3)	22(1)
C(53)	3961(5)	5226(4)	-309(3)	29(1)
C(54)	5175(4)	4877(4)	130(3)	31(1)
C(55)	5059(4)	4592(4)	1150(4)	33(1)
C(56)	3716(4)	4652(4)	1750(3)	24(1)
C(57)	884(4)	4844(3)	3205(3)	16(1)
C(58)	1522(4)	5578(3)	3644(3)	19(1)
C(59)	1483(4)	5539(3)	4674(3)	21(1)
C(60)	774(4)	4751(4)	5324(3)	22(1)
C(61)	101(4)	4036(3)	4926(3)	22(1)
C(62)	152(4)	4084(3)	3887(3)	19(1)

C(63)	345(4)	3875(3)	1628(3)	19(1)
C(64)	-485(4)	3961(4)	881(3)	22(1)
C(65)	-840(5)	3004(4)	557(3)	27(1)
C(66)	-392(5)	1881(4)	968(3)	29(1)
C(67)	457(4)	1737(4)	1710(3)	27(1)
C(68)	823(4)	2711(3)	2012(3)	21(1)
C(41A)	-127(10)	7520(11)	8680(9)	29(3)
C(42A)	-463(14)	6586(11)	8121(9)	49(4)
C(43A)	-1885(17)	6235(17)	8610(11)	51(4)
C(41B)	52(9)	7527(7)	7984(7)	25(2)
C(42B)	-515(9)	6931(9)	8998(7)	29(3)
C(43B)	-1997(12)	6629(12)	8995(9)	32(3)
B(1)	4999(4)	2946(4)	6566(3)	17(1)
B(2)	889(4)	4987(4)	1976(3)	16(1)

Table 3. Bond lengths [Å] and angles [°] for dm.

Cl(1)-C(2)	1.694(4)
Cl(2)-C(3)	1.694(4)
Cl(3)-C(36)	1.696(4)
Cl(4)-C(37)	1.698(4)
N(1)-C(1)	1.328(5)
N(1)-C(3)	1.381(5)
N(1)-C(4)	1.477(5)
N(2)-C(1)	1.315(5)
N(2)-C(2)	1.385(5)
N(2)-C(10)	1.469(5)
N(3)-C(35)	1.324(5)
N(3)-C(36)	1.373(5)
N(3)-C(38)	1.469(5)
N(4)-C(35)	1.337(5)
N(4)-C(37)	1.364(5)
N(4)-C(44)	1.469(5)
C(2)-C(3)	1.336(5)
C(4)-C(5)	1.525(5)
C(5)-C(6)	1.532(5)
C(6)-C(7)	1.534(5)
C(7)-C(8)	1.536(5)
C(8)-C(9)	1.528(6)
C(11)-C(12)	1.406(5)
C(11)-C(16)	1.409(5)
C(11)-B(1)	1.657(5)
C(12)-C(13)	1.397(6)
C(13)-C(14)	1.397(6)
C(14)-C(15)	1.386(7)
C(15)-C(16)	1.395(6)
C(17)-C(18)	1.400(5)
C(17)-C(22)	1.412(6)
C(17)-B(1)	1.649(6)
C(18)-C(19)	1.389(6)
C(19)-C(20)	1.367(7)

C(20)-C(21)	1.405(6)
C(21)-C(22)	1.381(6)
C(23)-C(24)	1.400(6)
C(23)-C(28)	1.412(6)
C(23)-B(1)	1.639(6)
C(24)-C(25)	1.376(6)
C(25)-C(26)	1.393(7)
C(26)-C(27)	1.381(7)
C(27)-C(28)	1.380(6)
C(29)-C(30)	1.397(5)
C(29)-C(34)	1.412(5)
C(29)-B(1)	1.644(6)
C(30)-C(31)	1.394(5)
C(31)-C(32)	1.387(6)
C(32)-C(33)	1.380(7)
C(33)-C(34)	1.392(6)
C(36)-C(37)	1.349(6)
C(38)-C(39)	1.521(6)
C(39)-C(40)	1.512(8)
C(40)-C(41B)	1.510(10)
C(40)-C(41A)	1.704(12)
C(45)-C(50)	1.408(5)
C(45)-C(46)	1.409(5)
C(45)-B(2)	1.641(6)
C(46)-C(47)	1.389(5)
C(47)-C(48)	1.382(6)
C(48)-C(49)	1.382(6)
C(49)-C(50)	1.388(5)
C(51)-C(52)	1.392(6)
C(51)-C(56)	1.401(5)
C(51)-B(2)	1.653(6)
C(52)-C(53)	1.390(6)
C(53)-C(54)	1.399(7)
C(54)-C(55)	1.376(7)
C(55)-C(56)	1.418(6)
C(57)-C(58)	1.401(5)

C(57)-C(62)	1.401(5)
C(57)-B(2)	1.659(5)
C(58)-C(59)	1.391(5)
C(59)-C(60)	1.391(6)
C(60)-C(61)	1.376(6)
C(61)-C(62)	1.400(5)
C(63)-C(64)	1.397(6)
C(63)-C(68)	1.410(5)
C(63)-B(2)	1.647(6)
C(64)-C(65)	1.378(6)
C(65)-C(66)	1.376(6)
C(66)-C(67)	1.398(6)
C(67)-C(68)	1.386(6)
C(41A)-C(42A)	1.523(16)
C(42A)-C(43A)	1.547(19)
C(41B)-C(42B)	1.526(13)
C(42B)-C(43B)	1.568(15)

C(1)-N(1)-C(3)	107.3(3)
C(1)-N(1)-C(4)	126.4(3)
C(3)-N(1)-C(4)	126.3(3)
C(1)-N(2)-C(2)	107.3(3)
C(1)-N(2)-C(10)	126.6(3)
C(2)-N(2)-C(10)	126.1(3)
C(35)-N(3)-C(36)	108.0(4)
C(35)-N(3)-C(38)	125.1(4)
C(36)-N(3)-C(38)	126.7(4)
C(35)-N(4)-C(37)	108.0(3)
C(35)-N(4)-C(44)	124.7(4)
C(37)-N(4)-C(44)	127.3(4)
N(2)-C(1)-N(1)	110.3(3)
C(3)-C(2)-N(2)	107.7(3)
C(3)-C(2)-Cl(1)	130.2(3)
N(2)-C(2)-Cl(1)	122.0(3)
C(2)-C(3)-N(1)	107.4(3)
C(2)-C(3)-Cl(2)	131.1(3)

N(1)-C(3)-Cl(2)	121.5(3)
N(1)-C(4)-C(5)	111.8(3)
C(4)-C(5)-C(6)	111.3(3)
C(5)-C(6)-C(7)	113.0(3)
C(6)-C(7)-C(8)	113.5(3)
C(9)-C(8)-C(7)	113.0(4)
C(12)-C(11)-C(16)	115.2(3)
C(12)-C(11)-B(1)	122.9(3)
C(16)-C(11)-B(1)	121.6(3)
C(13)-C(12)-C(11)	123.2(4)
C(12)-C(13)-C(14)	119.3(4)
C(15)-C(14)-C(13)	119.5(4)
C(14)-C(15)-C(16)	120.0(4)
C(15)-C(16)-C(11)	122.7(4)
C(18)-C(17)-C(22)	114.9(4)
C(18)-C(17)-B(1)	122.5(3)
C(22)-C(17)-B(1)	122.4(3)
C(19)-C(18)-C(17)	122.7(4)
C(20)-C(19)-C(18)	121.4(4)
C(19)-C(20)-C(21)	117.8(4)
C(22)-C(21)-C(20)	120.6(4)
C(21)-C(22)-C(17)	122.6(4)
C(24)-C(23)-C(28)	114.8(4)
C(24)-C(23)-B(1)	120.3(3)
C(28)-C(23)-B(1)	124.6(3)
C(25)-C(24)-C(23)	123.2(4)
C(24)-C(25)-C(26)	119.9(4)
C(27)-C(26)-C(25)	119.1(4)
C(28)-C(27)-C(26)	120.1(4)
C(27)-C(28)-C(23)	122.9(4)
C(30)-C(29)-C(34)	115.3(3)
C(30)-C(29)-B(1)	124.4(3)
C(34)-C(29)-B(1)	120.0(3)
C(31)-C(30)-C(29)	122.6(4)
C(32)-C(31)-C(30)	120.7(4)
C(33)-C(32)-C(31)	118.2(4)

C(32)-C(33)-C(34)	121.1(4)
C(33)-C(34)-C(29)	122.1(4)
N(3)-C(35)-N(4)	109.1(4)
C(37)-C(36)-N(3)	107.4(4)
C(37)-C(36)-Cl(3)	130.4(3)
N(3)-C(36)-Cl(3)	122.2(3)
C(36)-C(37)-N(4)	107.5(4)
C(36)-C(37)-Cl(4)	129.4(3)
N(4)-C(37)-Cl(4)	123.0(3)
N(3)-C(38)-C(39)	112.0(3)
C(40)-C(39)-C(38)	112.1(4)
C(41B)-C(40)-C(39)	125.5(6)
C(41B)-C(40)-C(41A)	33.0(4)
C(39)-C(40)-C(41A)	98.7(5)
C(50)-C(45)-C(46)	114.8(3)
C(50)-C(45)-B(2)	120.2(3)
C(46)-C(45)-B(2)	124.9(3)
C(47)-C(46)-C(45)	122.3(4)
C(48)-C(47)-C(46)	120.9(4)
C(47)-C(48)-C(49)	118.8(4)
C(48)-C(49)-C(50)	120.1(4)
C(49)-C(50)-C(45)	123.1(4)
C(52)-C(51)-C(56)	115.5(4)
C(52)-C(51)-B(2)	120.1(3)
C(56)-C(51)-B(2)	124.3(3)
C(53)-C(52)-C(51)	123.9(4)
C(52)-C(53)-C(54)	119.1(4)
C(55)-C(54)-C(53)	119.5(4)
C(54)-C(55)-C(56)	120.1(4)
C(51)-C(56)-C(55)	121.9(4)
C(58)-C(57)-C(62)	114.9(4)
C(58)-C(57)-B(2)	120.9(3)
C(62)-C(57)-B(2)	124.0(3)
C(59)-C(58)-C(57)	123.2(4)
C(60)-C(59)-C(58)	120.1(4)
C(61)-C(60)-C(59)	118.5(4)

C(60)-C(61)-C(62)	120.7(4)
C(61)-C(62)-C(57)	122.6(4)
C(64)-C(63)-C(68)	114.0(4)
C(64)-C(63)-B(2)	125.0(3)
C(68)-C(63)-B(2)	120.7(3)
C(65)-C(64)-C(63)	123.6(4)
C(66)-C(65)-C(64)	121.0(4)
C(65)-C(66)-C(67)	118.1(4)
C(68)-C(67)-C(66)	120.0(4)
C(67)-C(68)-C(63)	123.3(4)
C(42A)-C(41A)-C(40)	105.2(8)
C(41A)-C(42A)-C(43A)	110.6(12)
C(40)-C(41B)-C(42B)	107.3(7)
C(41B)-C(42B)-C(43B)	111.3(7)
C(23)-B(1)-C(29)	110.6(3)
C(23)-B(1)-C(17)	113.4(3)
C(29)-B(1)-C(17)	105.0(3)
C(23)-B(1)-C(11)	104.0(3)
C(29)-B(1)-C(11)	113.3(3)
C(17)-B(1)-C(11)	110.9(3)
C(45)-B(2)-C(63)	114.8(3)
C(45)-B(2)-C(51)	108.4(3)
C(63)-B(2)-C(51)	105.5(3)
C(45)-B(2)-C(57)	104.9(3)
C(63)-B(2)-C(57)	111.2(3)
C(51)-B(2)-C(57)	112.1(3)

Symmetry transformations used to generate equivalent atoms:

Table 4. Anisotropic displacement parameters ($Å^2$x 10^3) for dm. The anisotropic displacement factor exponent takes the form: $-2\pi^2[h^2 a^{*2}U^{11} + ... + 2 h k a^* b^* U^{12}]$

	U^{11}	U^{22}	U^{33}	U^{23}	U^{13}	U^{12}
Cl(1)	14(1)	29(1)	41(1)	-9(1)	-7(1)	-6(1)
Cl(2)	23(1)	12(1)	38(1)	-3(1)	-10(1)	0(1)
Cl(3)	28(1)	18(1)	40(1)	2(1)	-9(1)	-3(1)
Cl(4)	38(1)	31(1)	29(1)	-5(1)	-8(1)	-4(1)
N(1)	10(1)	17(2)	20(2)	-4(1)	-3(1)	1(1)
N(2)	22(2)	12(2)	16(2)	-3(1)	4(1)	-3(1)
N(3)	21(2)	22(2)	22(2)	-2(1)	6(1)	-3(1)
N(4)	28(2)	22(2)	18(2)	-3(1)	10(1)	-3(1)
C(1)	23(2)	19(2)	20(2)	-6(2)	-3(2)	2(2)
C(2)	11(2)	15(2)	23(2)	-8(2)	1(1)	0(1)
C(3)	15(2)	15(2)	18(2)	-4(2)	-1(1)	0(1)
C(4)	6(2)	30(2)	23(2)	-7(2)	-1(1)	-3(1)
C(5)	16(2)	27(2)	24(2)	-6(2)	0(2)	-2(2)
C(6)	21(2)	22(2)	27(2)	-12(2)	-4(2)	0(2)
C(7)	18(2)	20(2)	35(2)	-13(2)	-3(2)	-1(2)
C(8)	21(2)	26(2)	37(2)	-13(2)	-9(2)	-1(2)
C(9)	28(2)	28(2)	43(3)	-7(2)	-2(2)	-8(2)
C(10)	34(2)	16(2)	32(2)	-5(2)	1(2)	-8(2)
C(11)	16(2)	19(2)	15(2)	0(1)	-2(1)	-1(1)
C(12)	18(2)	25(2)	17(2)	-4(2)	2(2)	-1(2)
C(13)	25(2)	32(2)	23(2)	0(2)	0(2)	-9(2)
C(14)	19(2)	43(3)	34(2)	-4(2)	8(2)	-3(2)
C(15)	27(2)	32(2)	21(2)	-9(2)	9(2)	-1(2)
C(16)	21(2)	25(2)	16(2)	-3(2)	5(2)	-6(2)
C(17)	21(2)	19(2)	13(2)	-2(1)	5(1)	-4(2)
C(18)	24(2)	22(2)	22(2)	-2(2)	-1(2)	-1(2)
C(19)	36(2)	17(2)	32(2)	-2(2)	2(2)	-1(2)
C(20)	43(3)	22(2)	29(2)	-6(2)	9(2)	-11(2)
C(21)	24(2)	30(2)	30(2)	-9(2)	8(2)	-10(2)
C(22)	17(2)	22(2)	24(2)	-4(2)	7(2)	-2(2)
C(23)	11(2)	22(2)	19(2)	-5(2)	6(1)	-4(1)

C(24)	20(2)	29(2)	25(2)	-1(2)	-1(2)	-2(2)
C(25)	25(2)	25(2)	42(3)	2(2)	0(2)	2(2)
C(26)	19(2)	48(3)	38(3)	14(2)	-5(2)	3(2)
C(27)	25(2)	51(3)	31(2)	3(2)	-7(2)	-11(2)
C(28)	22(2)	30(2)	22(2)	-1(2)	0(2)	-6(2)
C(29)	16(2)	10(2)	19(2)	-4(1)	6(1)	-4(1)
C(30)	23(2)	14(2)	20(2)	-6(2)	4(2)	-2(1)
C(31)	34(2)	17(2)	23(2)	3(2)	-4(2)	-5(2)
C(32)	48(3)	16(2)	21(2)	-7(2)	15(2)	-6(2)
C(33)	31(2)	18(2)	30(2)	0(2)	14(2)	-5(2)
C(34)	15(2)	16(2)	26(2)	-5(2)	5(2)	0(1)
C(35)	31(2)	20(2)	21(2)	-5(2)	6(2)	-2(2)
C(36)	19(2)	21(2)	23(2)	2(2)	2(2)	0(2)
C(37)	11(2)	32(2)	21(2)	-5(2)	5(2)	0(2)
C(38)	25(2)	21(2)	25(2)	-4(2)	4(2)	-6(2)
C(39)	22(2)	32(2)	41(3)	7(2)	3(2)	0(2)
C(40)	37(3)	32(3)	81(4)	4(3)	-29(3)	-12(2)
C(44)	48(3)	18(2)	28(2)	-1(2)	6(2)	-1(2)
C(45)	19(2)	18(2)	11(2)	-6(1)	2(1)	-2(1)
C(46)	19(2)	21(2)	18(2)	-3(2)	2(2)	-2(2)
C(47)	16(2)	32(2)	19(2)	-1(2)	2(2)	9(2)
C(48)	28(2)	21(2)	23(2)	-1(2)	-2(2)	9(2)
C(49)	32(2)	19(2)	26(2)	-7(2)	1(2)	-4(2)
C(50)	18(2)	17(2)	22(2)	-9(2)	1(2)	-2(1)
C(51)	17(2)	12(2)	29(2)	-5(2)	0(2)	-1(1)
C(52)	20(2)	11(2)	33(2)	0(2)	-2(2)	0(1)
C(53)	36(3)	21(2)	26(2)	-8(2)	11(2)	-7(2)
C(54)	15(2)	34(2)	42(2)	-17(2)	17(2)	-11(2)
C(55)	14(2)	35(3)	49(3)	-12(2)	-5(2)	-2(2)
C(56)	12(2)	28(2)	30(2)	-3(2)	0(2)	-1(2)
C(57)	10(2)	13(2)	24(2)	-2(2)	0(1)	2(1)
C(58)	13(2)	17(2)	24(2)	-1(2)	0(2)	0(1)
C(59)	16(2)	23(2)	25(2)	-7(2)	-10(2)	1(2)
C(60)	20(2)	25(2)	16(2)	-1(2)	-3(2)	6(2)
C(61)	18(2)	23(2)	20(2)	4(2)	0(2)	5(2)
C(62)	17(2)	13(2)	25(2)	-4(2)	-3(2)	2(1)

C(63)	15(2)	22(2)	18(2)	-6(2)	7(1)	-4(1)
C(64)	18(2)	23(2)	21(2)	-5(2)	7(2)	-3(2)
C(65)	25(2)	38(3)	20(2)	-7(2)	2(2)	-11(2)
C(66)	31(2)	26(2)	31(2)	-11(2)	5(2)	-12(2)
C(67)	27(2)	21(2)	29(2)	-5(2)	7(2)	-5(2)
C(68)	21(2)	18(2)	21(2)	-7(2)	7(2)	-1(2)
C(41A)	13(4)	41(7)	31(7)	-8(5)	-1(4)	2(4)
C(42A)	64(8)	46(7)	46(7)	2(5)	-11(6)	-33(6)
C(43A)	44(8)	83(13)	28(7)	6(7)	-9(6)	-26(8)
C(41B)	20(4)	27(5)	24(5)	8(4)	-5(3)	0(3)
C(42B)	21(4)	31(5)	32(5)	11(4)	1(4)	-10(4)
C(43B)	25(5)	49(7)	21(6)	10(5)	-1(5)	-15(5)
B(1)	13(2)	17(2)	20(2)	-1(2)	3(2)	-4(2)
B(2)	13(2)	18(2)	15(2)	0(2)	-2(2)	-1(2)

Table 5. Hydrogen coordinates (x 10^4) and isotropic displacement parameters ($Å^2$x 10^3) for dm.

	x	y	z	U(eq)
H(10)	8514	5959	4242	26
H(13A)	8754	9021	3832	23
H(13B)	9685	7772	4113	23
H(14A)	8762	7832	5812	27
H(14B)	7619	8973	5578	27
H(15A)	9464	9540	6190	28
H(15B)	9411	10115	5062	28
H(16A)	11293	8579	4490	29
H(16B)	11374	8075	5631	29
H(17A)	12015	9880	5915	32
H(17B)	13158	9163	5122	32
H(18A)	12124	10439	3796	49
H(18B)	12844	11112	4458	49
H(18C)	11175	11215	4640	49
H(19A)	6092	5102	3158	41
H(19B)	5375	5038	4311	41
H(19C)	7014	4542	4015	41
H(33)	3166	1537	6195	25
H(34)	866	1340	6860	32
H(35)	-419	2552	8090	41
H(36)	626	3952	8620	34
H(37)	2922	4140	7943	26
H(43)	3376	5184	6279	28
H(42)	3825	7073	6155	36
H(41)	5977	7419	6401	38
H(40)	7781	5784	6633	34
H(39)	7346	3893	6741	27
H(25)	5830	549	6659	31
H(24)	6791	-868	7805	39
H(23)	7440	-321	9233	45

H(22)	7112	1672	9469	43
H(21)	6090	3082	8339	30
H(27)	3699	3037	4855	24
H(28)	4576	2711	3196	30
H(29)	6995	2155	2675	36
H(30)	8485	1811	3877	35
H(31)	7606	2059	5550	24
H(1)	2925	11347	8551	31
H(6A)	3867	8240	8439	29
H(6B)	3221	9408	7801	29
H(7A)	1599	8257	9457	41
H(7B)	981	9356	8734	41
H(9A)	3770	12890	9443	50
H(9B)	4067	12495	10566	50
H(9C)	2491	12780	10338	50
H(57)	-1998	5825	1825	24
H(58)	-3287	7699	1581	30
H(59)	-2216	9350	1376	31
H(60)	191	9091	1417	31
H(61)	1505	7217	1579	23
H(67)	1846	5494	-14	27
H(66)	4024	5432	-1012	34
H(65)	6073	4839	-273	37
H(64)	5879	4354	1454	39
H(63)	3654	4459	2454	29
H(45)	2004	6131	3217	23
H(46)	1942	6050	4934	25
H(47)	754	4708	6028	26
H(48)	-404	3503	5359	27
H(49)	-327	3583	3634	22
H(55)	-823	4722	580	26
H(54)	-1403	3122	42	32
H(53)	-652	1222	753	35
H(52)	783	972	2008	32
H(51)	1426	2588	2502	25

1-(2-naphthylmethyl)-3-methyl-4,5-dichloroimidazolium iodide (C).

1-(2-naphthylmethyl)-4,5-dichloroimidazole (0.856 g, 0.003 mol) was dissolved in 7mL of CH_3CN at reflux. Methyl iodide (3 mL, 0.043 mol) was added and the solution was allowed to reflux for 10h. The volatiles were removed in vacuo yielding a yellow solid. The solid was washed with acetone to give a light yellow solid (0.962 g, 76%). ^{1}H NMR (300MHz, d_6-DMSO) δ 3.85 (s), 5.68 (s), 7.57 (m), 7.96 (m), 9.50 (s). ^{13}C NMR {^{1}H} (75MHz, d_6-DMSO) δ 35.89, 52.17, 118.98, 126.25, 127.50, 127.58, 128.26, 128.41, 128.56, 129.50, 130.85, 133.37, 133.47, 137.43.

Synthesis of (1,3-dimethyl-4,5-dichloroimidazole-2-ylidene)silver(I)acetate (1). Compound **A** (1.51 g, 5.15 mmol) was dissolved in dichloromethane (50 mL) and silver acetate (1.78 g, 10.7 mmol) was added. The mixture was stirred at room temperature for 2.5 h. The yellow precipitate, presumably AgI, was filtered and discarded. The volume of the reaction mixture was reduced under pressure to 5 mL. Hexane (1 L) was added and the fine white precipitate was filtered and washed with 20 mL of hexane. Yield: (1.35 g, 4.07 mmol, 79%); Mp: 187-188 ˚C. Anal. Calc. for $C_7H_9AgCl_2N_2O_2$: C, 25.33; H, 2.73; N, 8.44. Found: C, 25.37; H, 2.69; N, 8.01. ESI-MS (m/z): calc., 165.0, [$C_5H_7Cl_2N_2^+$], 272.8 [$C_5H_6AgCl_2N_2^+$], 436.8 [$C_{10}H_{12}AgCl_4N_4$]; found, 164.9, 272.7, and 436.7. ^{1}H NMR (300 MHz, DMSO-d_6): δ 1.79 (s, 3H, $COCH_3$), 3.77 (s, 6H, N-CH_3). ^{13}C {^{1}H} NMR (75 MHz, DMSO-d_6): δ 179.7 (C-Ag), 175.7 (C=O), 116.9 (C-Cl), 37.5 (N-CH_3), 23.4 ($COCH_3$). ^{109}Ag (35 MHz, DMSO- d_6): δ 352.55 (s, C-Ag-O).

1-hexyl-3-methyl-4,5-dichloroimidazole-AgOAc (2). B (2.20 g, 0.006 mol) was dissolved in 50 mL of CH_2Cl_2 and AgOAc (2.03 g, 0.012 mol) was added. The solution was stirred at RT for 3h resulting in a yellow AgI precipitate. The AgI was filtered and the CH_2Cl_2 was removed in vacuo. The pale yellow solid was dissolved in 3 mL of CH_2Cl_2 and hexanes was added. The

solution was cooled in an ice bath and the off-white product precipitated and was filtered (2.15 g, 88%). ^{1}H NMR (300MHz d_6-DMSO) δ 0.85 (t), 1.27 (s), 1.71 (t), 1.84 (s), 3.78 (s), 4.14 (t). ^{13}C NMR {^{1}H} (75MHz, d_6-DMSO)

δ 13.82, 21.93, 22.31, 25.28, 29.99, 30.65, 38.67, 50.30, 116.17, 117.10, 174.04, 179.64. Anal. Calcd. for $C_{12}H_{19}N_2O_2Ag_1Cl_2$ Theoretical: C, 35.85; H, 4.76; N, 6.97. Found: C, 35.12; H, 4.47, N, 6.69. MS-ESI m/z 343.

1-(2-napthylmethyl)-3-methyl-4,5-dichloroimidazole-AgOAc (3). C (0.942 g, 0.002 mol) was added to CH_2Cl_2 and stirred. Silver acetate (0.751 g, 0.004 mol) was added and silver iodide formed immediately. The solution was stirred for 3h after which the precipitate was filtered and solvent removed in vacuo. The resulting yellow oily solid was stirred in Et_2O yielding a white solid (0.772 g, 75%). ^{1}H NMR (300MHz d_6-DMSO)

δ 1.78 (s), 3.79 (s), 5.57 (s), 7.49 (m), 7.88 (m). ^{13}C NMR {^{1}H} (75MHz, d_6-DMSO)

δ 23.30, 37.72, 53.59, 116.48, 117.70, 125.10, 126.38, 126.46, 126.47, 127.52, 127.79, 128.47, 132.47, 132.70, 132.87, 175.70, 180.47. Anal. Calcd. for $C_{17}H_{15}N_2O_2AgCl_2$ Theoretical: C, 43.1; H, 3.4; N, 6.27. Found: C, 43.7; H, 3.3; N, 5.8. MS-ESI m/z 691.7.

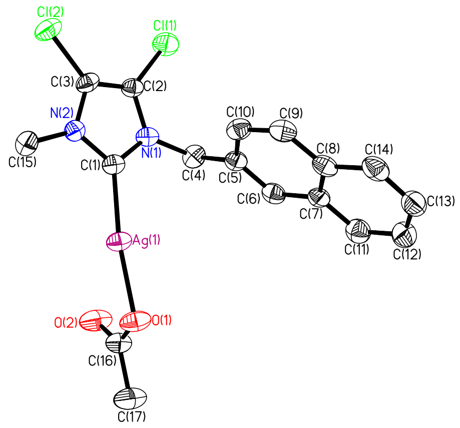

Crystal Structure of 3.

Table 1. Crystal data and structure refinement for **3**.

Identification code	$\mathbf{C_{17}H_{15}AgCl_2N_2O_2}$	
Empirical formula	C17 H15 Ag Cl2 N2 O2	
Formula weight	458.08	
Temperature	100(2) K	
Wavelength	0.71073 Å	
Crystal system	Monoclinic	
Space group	C2/c	
Unit cell dimensions	a = 14.577(3) Å	α= 90°.
	b = 21.126(4) Å	β= 105.386(3)°.
	c = 11.540(2) Å	γ = 90°.
Volume	3426.6(11) $Å^3$	
Z	8	
Density (calculated)	1.776 mg/m^3	
Absorption coefficient	1.500 mm^{-1}	
F(000)	1824	
Crystal size	0.20 x 0.09 x 0.05 mm^3	
Theta range for data collection	1.74 to 26.30°.	
Index ranges	$-18 \leq h \leq 18$, $-26 \leq k \leq 26$, $-14 \leq l \leq 14$	
Reflections collected	13367	
Independent reflections	3471 [R(int) = 0.0393]	
Completeness to theta = 26.30°	100.0 %	
Absorption correction	Semi-empirical from equivalents	
Max. and min. transmission	0.9288 and 0.7535	
Refinement method	Full-matrix least-squares on F^2	
Data / restraints / parameters	3471 / 0 / 220	
Goodness-of-fit on F^2	1.268	
Final R indices [I>2sigma(I)]	R1 = 0.0524, wR2 = 0.1077	
R indices (all data)	R1 = 0.0600, wR2 = 0.1106	
Largest diff. peak and hole	1.148 and -0.924 e.$Å^{-3}$	

Table 2. Atomic coordinates (x 10^4) and equivalent isotropic displacement parameters ($Å^2$x 10^3) for **$C_{17}H_{15}AgCl_2N_2O_2$**. U(eq) is defined as one third of the trace of the orthogonalized U^{ij} tensor.

	x	y	z	U(eq)
Ag(1)	2148(1)	2824(1)	8630(1)	33(1)
Cl(1)	-1331(1)	1984(1)	9641(1)	49(1)
Cl(2)	-79(1)	636(1)	9206(1)	45(1)
O(1)	3188(3)	3500(2)	8505(3)	43(1)
O(2)	2377(3)	3440(2)	6611(3)	56(1)
N(1)	254(3)	2443(2)	9105(3)	29(1)
N(2)	1026(3)	1610(2)	8855(3)	29(1)
C(1)	1071(3)	2256(2)	8867(4)	29(1)
C(2)	-273(3)	1929(2)	9274(4)	30(1)
C(3)	197(4)	1412(2)	9114(4)	32(1)
C(4)	12(4)	3111(2)	9255(5)	37(1)
C(5)	521(3)	3372(2)	10488(4)	32(1)
C(6)	642(3)	4011(2)	10623(5)	34(1)
C(7)	1053(3)	4285(2)	11781(5)	32(1)
C(8)	1336(3)	3885(3)	12778(5)	35(1)
C(9)	1227(3)	3228(3)	12617(5)	37(1)
C(10)	827(4)	2977(2)	11500(5)	36(1)
C(11)	1175(4)	4945(3)	11930(5)	43(1)
C(12)	1555(4)	5193(3)	13044(5)	47(1)
C(13)	1839(4)	4789(3)	14052(5)	44(1)
C(14)	1737(4)	4159(3)	13949(5)	41(1)
C(15)	1788(4)	1197(2)	8705(5)	37(1)
C(16)	3018(4)	3675(3)	7426(5)	38(1)
C(17)	3629(4)	4202(3)	7150(5)	56(2)

Table 3. Bond lengths [Å] and angles [°] for $\mathbf{C_{17}H_{15}AgCl_2N_2O_2}$.

Ag(1)-C(1)	2.051(5)
Ag(1)-O(1)	2.117(3)
Ag(1)-Ag(1)#1	3.3462(9)
Cl(1)-C(2)	1.708(5)
Cl(2)-C(3)	1.699(5)
O(1)-C(16)	1.258(6)
O(2)-C(16)	1.241(6)
N(1)-C(1)	1.349(6)
N(1)-C(2)	1.375(6)
N(1)-C(4)	1.477(6)
N(2)-C(1)	1.365(6)
N(2)-C(3)	1.383(6)
N(2)-C(15)	1.459(6)
C(2)-C(3)	1.327(7)
C(4)-C(5)	1.523(7)
C(5)-C(6)	1.363(7)
C(5)-C(10)	1.408(7)
C(6)-C(7)	1.434(7)
C(7)-C(8)	1.398(7)
C(7)-C(11)	1.411(7)
C(8)-C(9)	1.405(7)
C(8)-C(14)	1.443(7)
C(9)-C(10)	1.372(7)
C(11)-C(12)	1.363(8)
C(12)-C(13)	1.414(8)
C(13)-C(14)	1.340(8)
C(16)-C(17)	1.511(7)
C(1)-Ag(1)-O(1)	172.66(17)
C(1)-Ag(1)-Ag(1)#1	71.89(12)
O(1)-Ag(1)-Ag(1)#1	107.14(9)
C(16)-O(1)-Ag(1)	108.0(3)
C(1)-N(1)-C(2)	110.7(4)
C(1)-N(1)-C(4)	123.8(4)

C(2)-N(1)-C(4)	125.3(4)
C(1)-N(2)-C(3)	110.0(4)
C(1)-N(2)-C(15)	124.2(4)
C(3)-N(2)-C(15)	125.5(4)
N(1)-C(1)-N(2)	104.6(4)
N(1)-C(1)-Ag(1)	127.1(3)
N(2)-C(1)-Ag(1)	128.3(3)
C(3)-C(2)-N(1)	107.5(4)
C(3)-C(2)-Cl(1)	128.6(4)
N(1)-C(2)-Cl(1)	123.9(4)
C(2)-C(3)-N(2)	107.1(4)
C(2)-C(3)-Cl(2)	130.2(4)
N(2)-C(3)-Cl(2)	122.7(4)
N(1)-C(4)-C(5)	112.6(4)
C(6)-C(5)-C(10)	119.2(5)
C(6)-C(5)-C(4)	118.8(4)
C(10)-C(5)-C(4)	121.9(4)
C(5)-C(6)-C(7)	121.0(5)
C(8)-C(7)-C(11)	119.8(5)
C(8)-C(7)-C(6)	118.8(4)
C(11)-C(7)-C(6)	121.4(5)
C(7)-C(8)-C(9)	119.3(5)
C(7)-C(8)-C(14)	119.1(5)
C(9)-C(8)-C(14)	121.6(5)
C(10)-C(9)-C(8)	120.7(5)
C(9)-C(10)-C(5)	120.9(5)
C(12)-C(11)-C(7)	120.1(5)
C(11)-C(12)-C(13)	120.0(5)
C(14)-C(13)-C(12)	121.7(5)
C(13)-C(14)-C(8)	119.2(5)
O(2)-C(16)-O(1)	122.9(5)
O(2)-C(16)-C(17)	120.2(5)
O(1)-C(16)-C(17)	116.9(5)

Symmetry transformations used to generate equivalent atoms:

#1 -x+1/2,-y+1/2,-z+2

Table 4. Anisotropic displacement parameters ($Å^2$x 10^3) for $\mathbf{C_{17}H_{15}AgCl_2N_2O_2}$. The anisotropic displacement factor exponent takes the form: $-2\pi^2[h^2 a^{*2}U^{11} + ... + 2 h k a^* b^* U^{12}]$

	U^{11}	U^{22}	U^{33}	U^{23}	U^{13}	U^{12}
Ag(1)	32(1)	35(1)	31(1)	7(1)	4(1)	-7(1)
Cl(1)	34(1)	53(1)	61(1)	-5(1)	15(1)	-8(1)
Cl(2)	54(1)	32(1)	47(1)	1(1)	9(1)	-13(1)
O(1)	39(2)	45(2)	39(2)	15(2)	1(2)	-12(2)
O(2)	57(3)	70(3)	38(2)	6(2)	5(2)	-29(2)
N(1)	25(2)	34(2)	25(2)	5(2)	1(2)	-3(2)
N(2)	31(2)	30(2)	25(2)	-1(2)	4(2)	-2(2)
C(1)	28(2)	34(3)	21(2)	3(2)	0(2)	-5(2)
C(2)	27(2)	34(2)	28(2)	1(2)	4(2)	-6(2)
C(3)	39(3)	29(2)	24(2)	2(2)	1(2)	-9(2)
C(4)	34(3)	34(3)	38(3)	5(2)	3(2)	6(2)
C(5)	19(2)	38(3)	40(3)	-1(2)	8(2)	-2(2)
C(6)	26(3)	40(3)	39(3)	6(2)	11(2)	6(2)
C(7)	20(2)	36(3)	43(3)	-2(2)	14(2)	0(2)
C(8)	22(2)	46(3)	40(3)	3(2)	14(2)	2(2)
C(9)	31(3)	43(3)	38(3)	9(2)	9(2)	0(2)
C(10)	30(3)	34(3)	44(3)	2(2)	9(2)	-3(2)
C(11)	42(3)	43(3)	48(3)	3(3)	16(3)	5(2)
C(12)	45(3)	47(3)	51(3)	-6(3)	18(3)	0(3)
C(13)	40(3)	54(4)	40(3)	-6(3)	15(2)	-5(3)
C(14)	26(3)	62(4)	36(3)	-2(3)	9(2)	-4(2)
C(15)	38(3)	32(3)	40(3)	-3(2)	8(2)	0(2)
C(16)	33(3)	43(3)	38(3)	5(2)	8(2)	-6(2)
C(17)	53(4)	66(4)	46(4)	8(3)	8(3)	-24(3)

Table 5. Hydrogen coordinates (x 10^4) and isotropic displacement parameters ($Å^2$x 10^3) for $\mathbf{C_{17}H_{15}AgCl_2N_2O_2}$.

	x	y	z	U(eq)
H(4A)	-684	3148	9138	44
H(4B)	183	3370	8629	44
H(6)	452	4278	9939	41
H(9)	1431	2954	13289	45
H(10)	756	2532	11409	43
H(11)	992	5217	11252	52
H(12)	1629	5638	13144	56
H(13)	2108	4968	14822	53
H(14)	1928	3896	14639	49
H(15A)	2274	1450	8474	56
H(15B)	1527	884	8076	56
H(15C)	2075	978	9464	56
H(17A)	3645	4173	6308	84
H(17B)	4276	4163	7674	84
H(17C)	3361	4612	7289	84

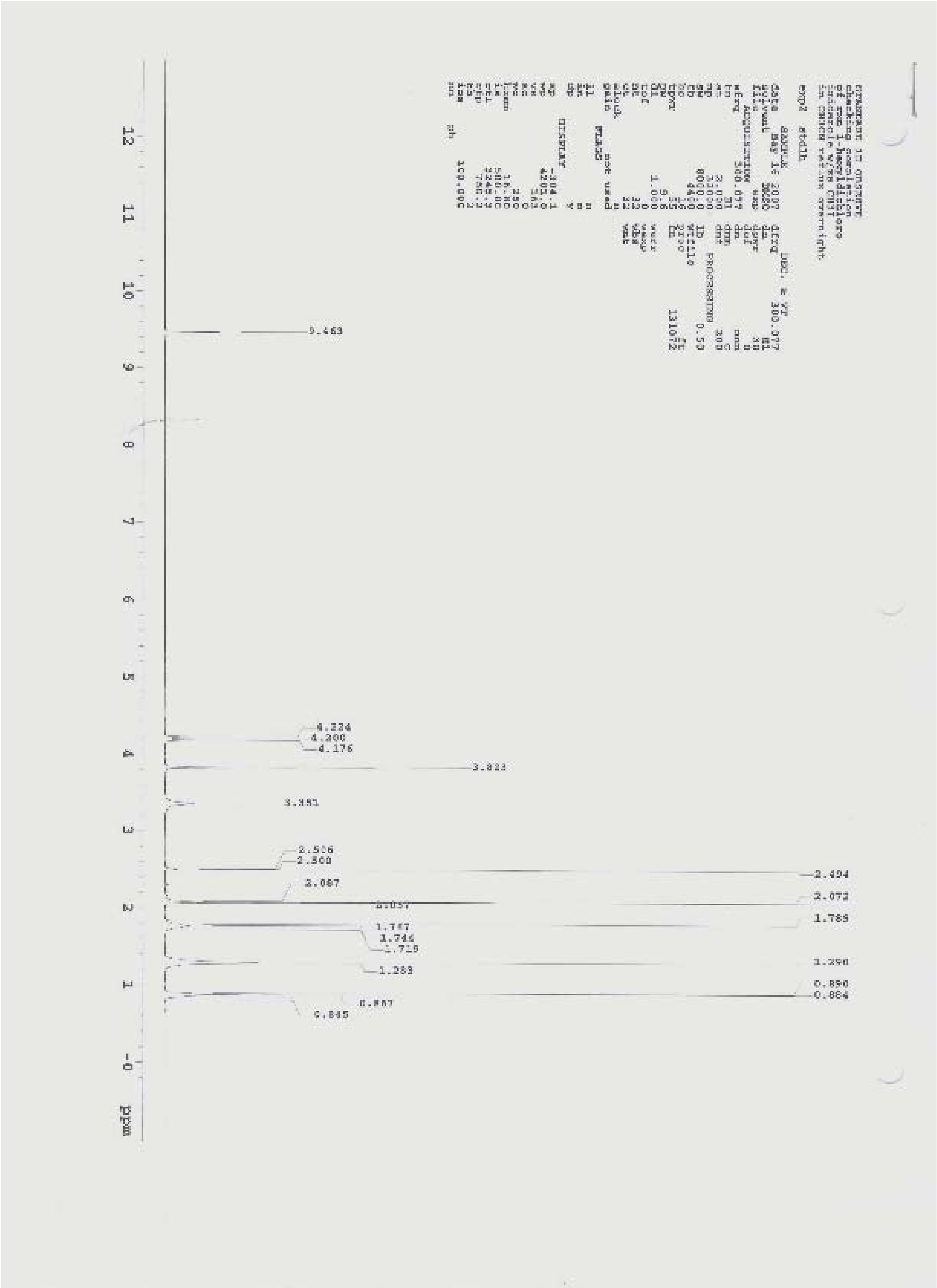

^{1}H NMR of imidazolium salt of B.

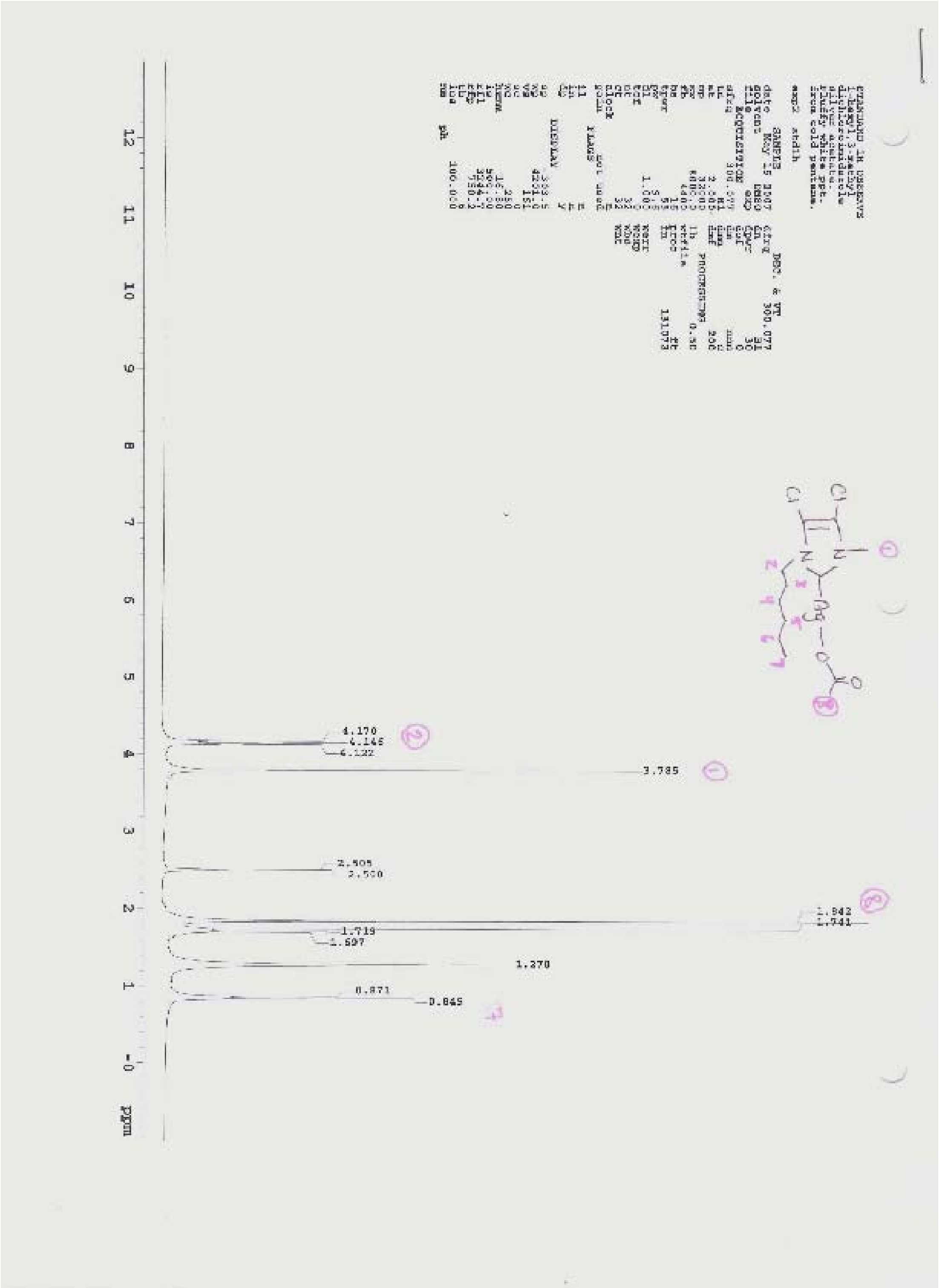
4.170
4.146
4.122
3.785
2.505
2.500
1.842
1.741
1.719
1.697
1.370
0.871
0.845
ppm

^1H NMR of **2**.

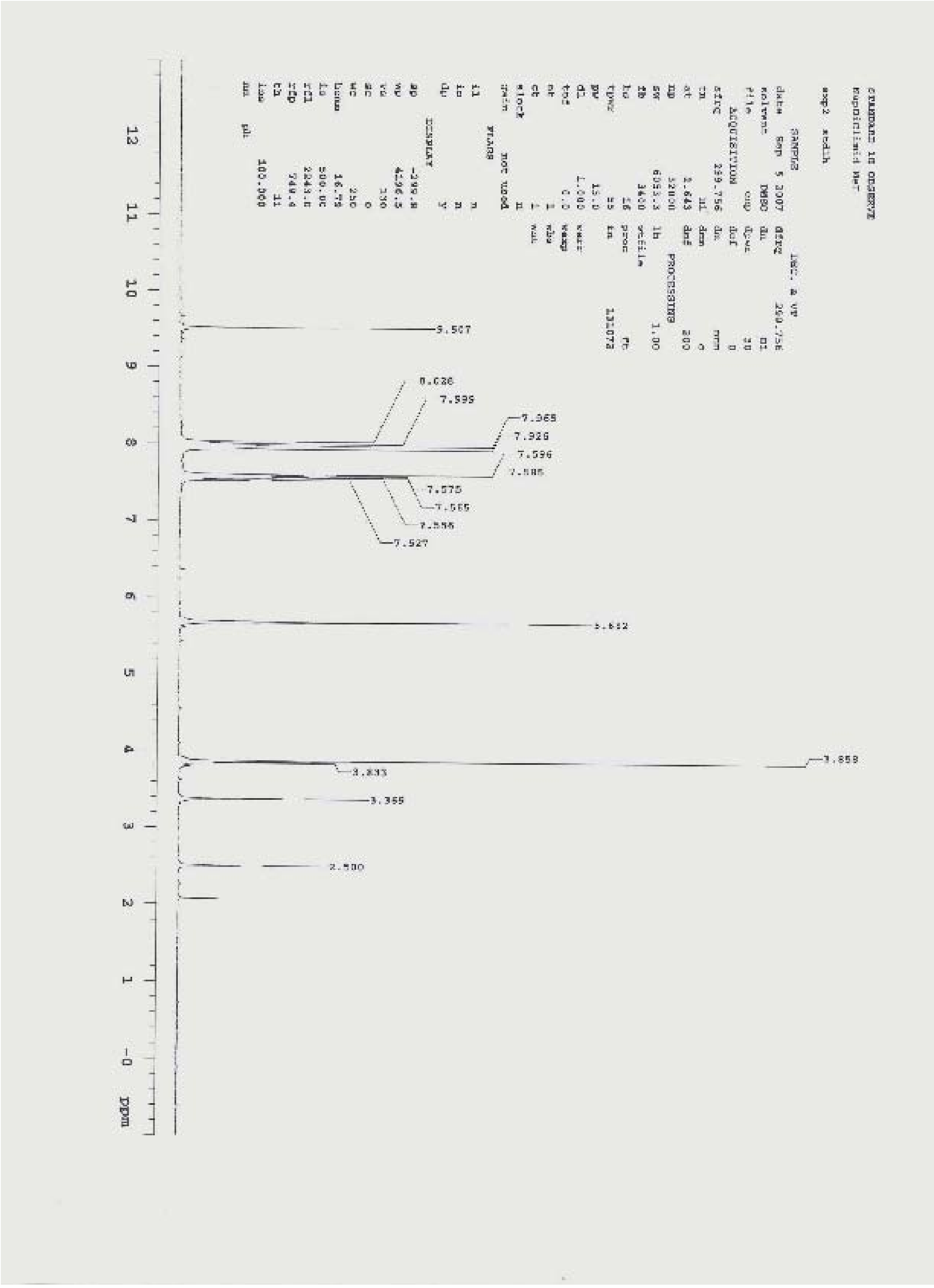

^{1}H NMR of imidazolium salt of C.

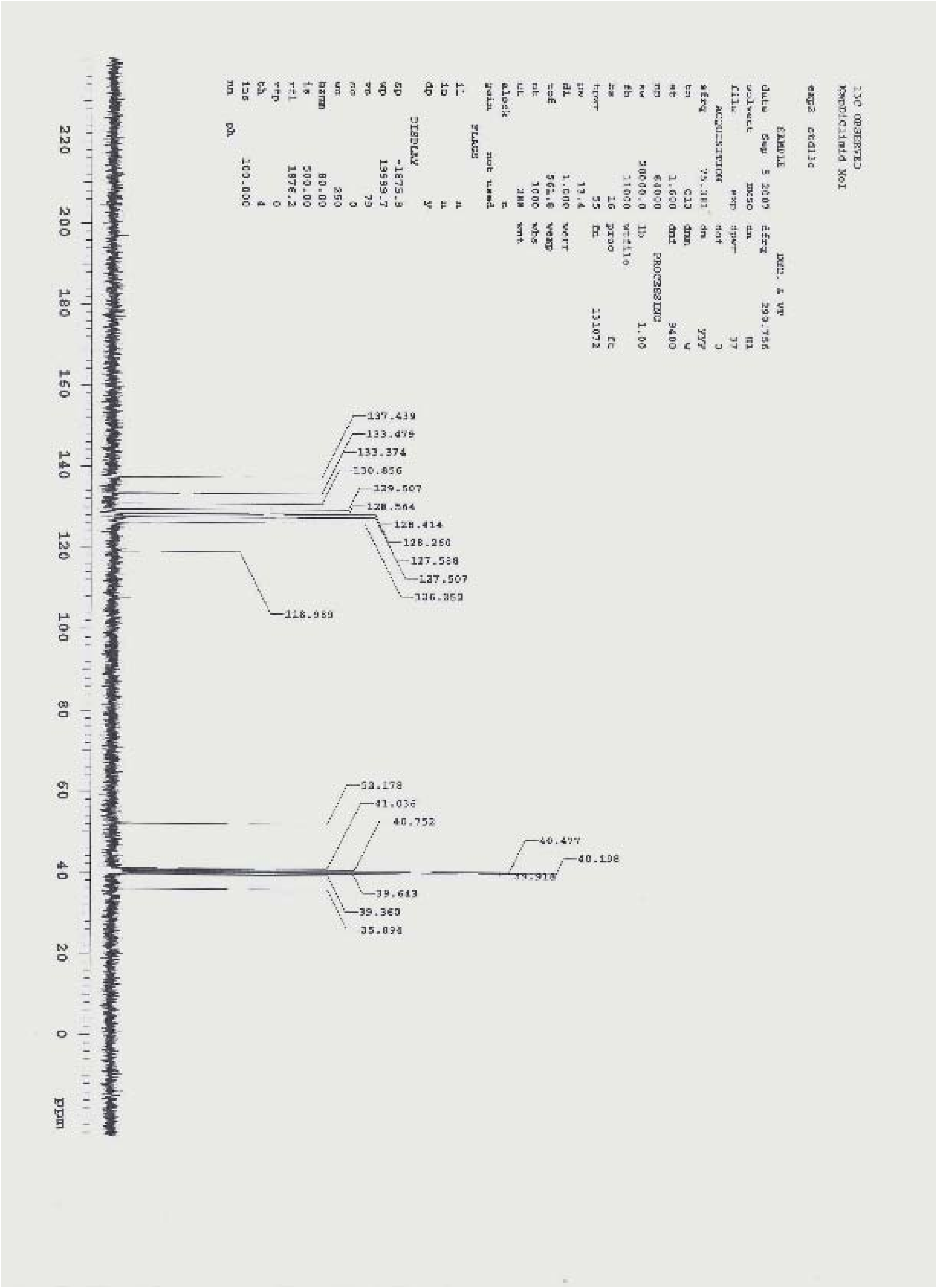

^{13}C NMR of imidazolium salt of C.

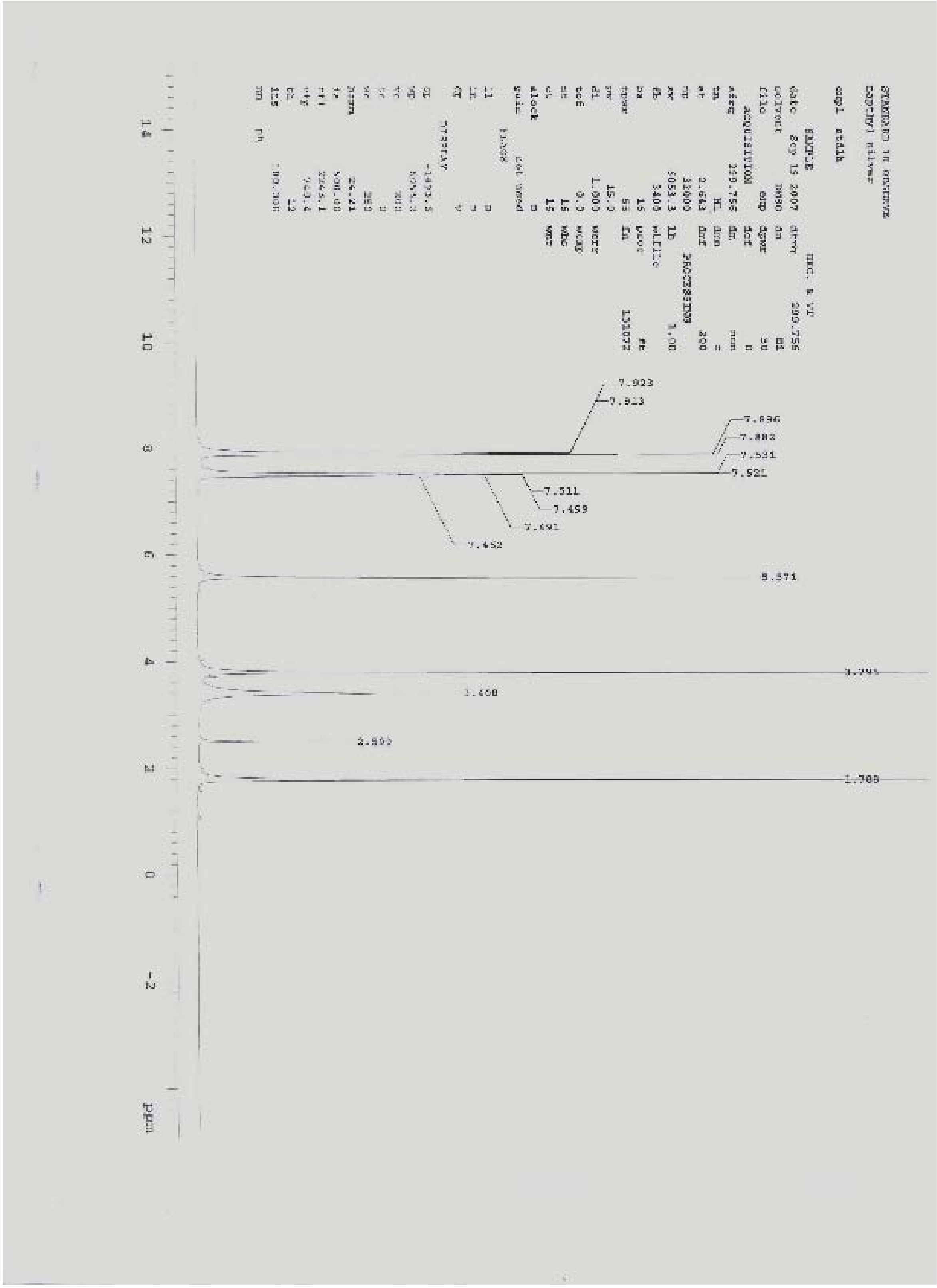

^{1}H NMR of **3**.

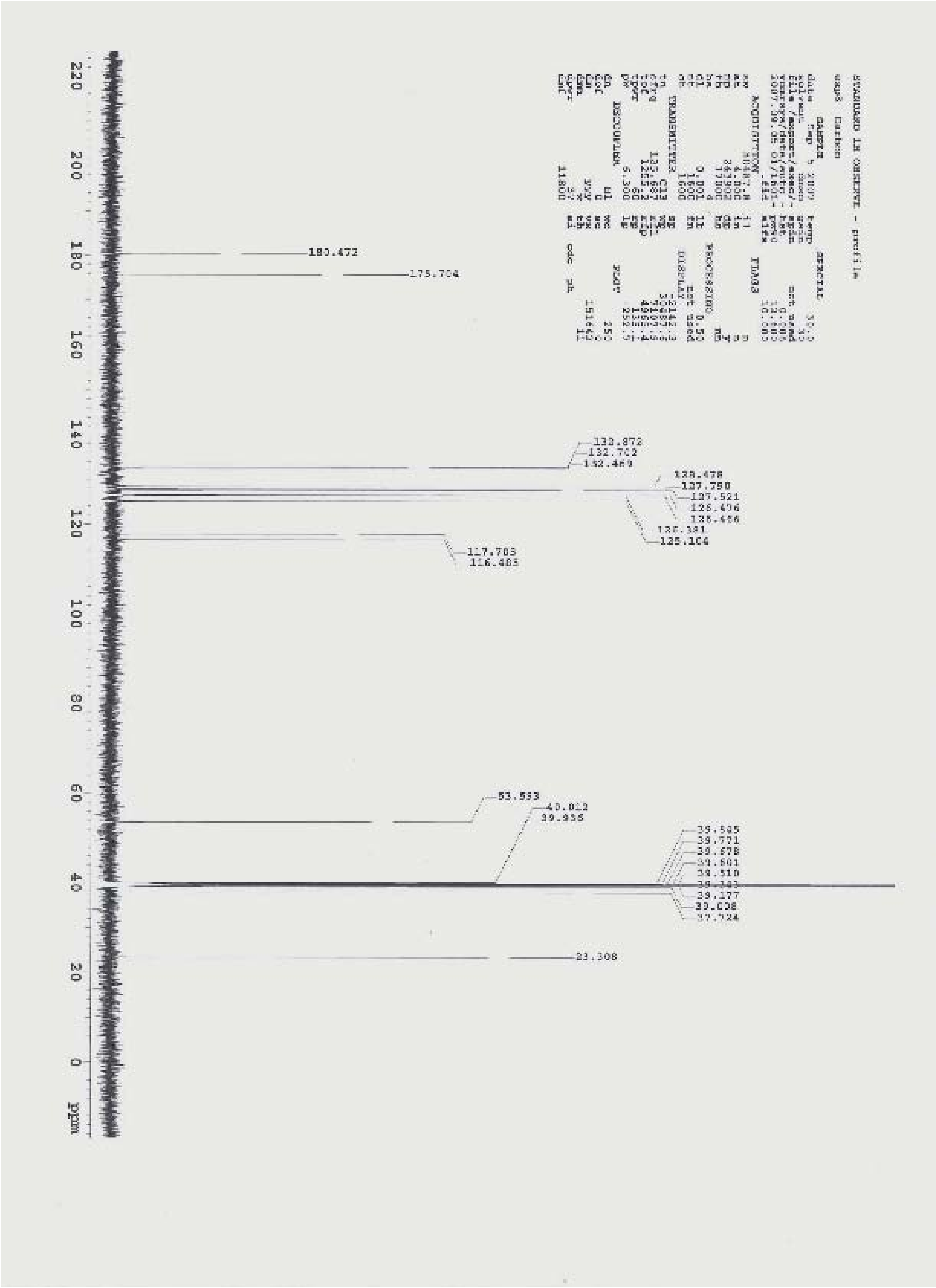

^{13}C NMR of **3**.
